# Docetaxel-Loaded Mixed Micelles and Polymersomes Composed of Poly (caprolactone)-Poly (ethylene glycol) (PEG-PCL) and Poly (lactic acid)-Poly (ethylene glycol) (PEG-PLA): Preparation and In-vitro Characterization

**Published:** 2019

**Authors:** Elham Khodaverdi, Zahra Tayarani-Najaran, Elham Minbashi, Mona Alibolandi, Javad Hosseini, Samaneh Sepahi, Hossein Kamali, Farzin Hadizadeh

**Affiliations:** a ***Targeted Drug Delivery Research Center, Pharmaceutical Technology Institute, Mashhad University of Medical Sciences, Mashhad, Iran. ***; b ***Department of Pharmacolgy, School of Pharmacy, Mashhad University of Medical Sciences, Mashhad, Iran. ***; c ***Pharmaceutical Research Center, Pharmaceutical Technology Institute, Mashhad University of Medical Sciences, Mashhad, Iran. ***; d ***Biotechnology Research Center, Pharmaceutical Technology Institute, Mashhad University of Medical Sciences, Mashhad, Iran.***

**Keywords:** Micelles, Polymersomes, Di-block copolymer, Docetaxel, C ytotoxicity

## Abstract

Microwave irradiation was used to synthesize poly (caprolactone)-poly (ethylene glycol) (PEG-PCL) and poly (lactic acid)-poly (ethylene glycol) (PEG-PLA) copolymers that are composed of biodegradable polymers including PEG, PLA, and PCL. These copolymers were used for loading docetaxel in nanoparticles. Single emulsion-solvent evaporation technique was applied for preparing the PEG-PLA and PEG-PCL mixed nanoparticles (micelles and polymersomes) with different proportions, including 0:1, 1:1, 3:1, 1:3, and 1:0. The unimodal gel permeation chromatography curve showed low polydispersity of the di-block copolymers. The *in-vitro* drug release curves of formulations were compared. Micelles and polymersomes of 75% PEG-PCL and 25% PEG-PLA (P5 and M5) have the lowest burst release (5%) at the same period compared to the other copolymers. The dynamic light scattering and TEM results clarified that the size and shape of the formulations are uniform. The cytotoxicity effect of P5 and M5 was evaluated in different cell lines. The best one was found to P5 with half maximal inhibitory concentration (IC_50_) between 1.48-11.79 µg/mL. The pro-apoptotic effect of P5 was confirmed with flow cytometry study. These mixed micelles (M5) and polymersomes (P5) was found to be superior formulations than non-mixed ones.

## Introduction

The self-assembling of block copolymers in the form of polymeric micelles and polymersomes has provided promising and desirable novel drug/gen delivery systems, especially in cancer treatment (1-3). Some important parameters related to these nanocarriers such as drug delivery, high tissue penetration, low toxicity, particle size, stability, release rate, release kinetics, and loading capacity could be modulated by changing the structure of block copolymers (4). Preparing these nanoparticles and loading drugs inside them is a fast and easy way to achieve solubilization of copolymer films and drugs in a water environment. PEG-PLA and PEG-PCL di-block are desirable materials because of their FDA approval, biocompatibility, biodegradability, low toxicity, and naturally degraded products (5). Plain PEG-PLA and PEG-PCL nanoparticles have been extensively used in drug delivery (6, 7). For example, Genexol^®^-PM is a micelle structure of paclitaxel made up of PLA-PEG copolymer that is in Phase IIc clinical trials and getting closer to being on the market (8). As mentioned above, one of the most valuable preferences of self-assembling structures compared to other nanoparticles is the possibility of optimizing of their critical characteristics, such as particle size, release rate, loading, and stability, by means of changing the structure of the block copolymers. However, that requires synthesis of a new copolymer with a modified structure. One of the best solutions to overcome this limitation and decrease numerous synthesis steps is to optimize a suitable block copolymer for nano self-assembling structures using mixed copolymers. By blending two or three types of block copolymers with different characteristics, a nano self-assembling structure with desirable characteristics can be achieved (9). This can provide the possibility of having multiple nanostructures with different characteristics just by a simple blending of two or three types of copolymers. For example, in a study by Zhao *et al*. curcumin was loaded in mixed micelles containing Pluronic P123 and Pluronic F68 prepared with different ratios, and the authors concluded that the mixed micelles created special properties, such as increased drug loading efficiency and micelle stability than individual components (3). In another study by Gao *et al*., a higher solubility of camptothecin and its greater toxicity against MCF-7 cancer cells *in-vitro* was achieved by mixed micelles composed of d-alpha-tocopheryl PEG 1,000 and Pluronic P105 (10). El-Dahmy *et al*. investigated increasing the *in-vivo* mean residence time of vinpocetine after intravenous injection using long circulating mixed micellar formulations (11) made of 32% w/w Pluronic F127 and 68% w/w Pluronic L121 as an optimum formula. Liu *et al*. showed a polymeric mixed micelle containing poly (lactide)-b-poly(N-isopropylacrylamide) (PLA-b-PNIPAM) and poly (lactide-b-poly(ethylene oxide) (PLA-b-PEG) prevented harmful protein aggregation, benefiting from the capture of thermally denatured proteins by PNIPAM followed by assisted refolding during cooling by PEG (9). As indicated above, many mixed polymeric micelles have been made and optimized for drug delivery, especially by means of different types of pluronics (12-14). However, PEG-PLA and PEG-PCL block copolymers were the most extensive materials used in constructing nano self-assembling structures such as polymeric micelles and polymersomes; mixed nanocarriers composed of these copolymers have not yet been studied. By blending these two copolymers, a desired formulation, benefitting from the privileges of both of them together, can be optimized; docetaxel delivery by means of mixed micelles and polymersomes of PEG-PCL and PEG-PLA and with different ratios was studied here. Docetaxel (DTX) is more efficient than paclitaxel in chemotherapy, but its limitations, such as poor solubility, rapid phagocytic clearance, and systemic toxicity, are the same as paclitaxel’s. To overcome these disadvantages, nanosized delivery systems are the best choice, as they control release rate, improve drug solubility, pharmacokinetics, and biodistribution, and reduce systemic side effects (1).

Our goal in this study was the characterization of such mixed nanostructures and determining the best formulation from the aspect of size, release rate, loading capacity, and cellular toxicity. We also evaluated and compared all of the above nanoparticle characteristics for both delivery systems, including micelles and polymersomes.

## Experimental

*Materials*

Methoxy-PEG (5000 Da), D,L-lactide, ε-Caprolactone, and Tin (II) 2-ethylhexanoate were purchased from Sigma-Aldrich (Darmstadt, Germany). DTX hydrochloride was obtained from Euroasia (New Delhi, India). All other materials and solvents were prepared from Merck (Darmstadt, Germany). 

*Preparation of PEG-PLA and PEG-PCL Copolymers*

The ring-opening copolymerization of PEG-PLA and PEG-PCL di-block was performed with different block lengths of PEG-PLA, 5,000:5,000 (LA5) and 5,000:15,000 (LA15), and PEG-PCL, 5,000:5,000 (CL5) and 5,000:15,000 (CL15), using microwave irradiation. For example, to synthesize the CL5 copolymer, the first 5.0 g of methoxy-PEG 5000 was loaded into a dry flask in a Milestone Microsynth microwave (Milestone, Italy) for 10 min at 1200 W and 130 °C to eliminate the water content of methoxy-PEG. After that, 4 g of D,L-lactide and 20 µL of Tin(II) 2-ethylhexanoate were loaded to the dried methoxy-PEG, and then stirred and irradiated for 120 min at 120 °C, 1000 W and 50 rpm. For purification, the di-block copolymers were dissolved in chloroform and then precipitated via diethyl ether. The residual solvent was removed at vacuum condition at 30 °C for 30 min using rotary evaporator. To eliminate the water residue, the products were freeze dried and maintained at a temperature of –20 °C until use. Di-block of PEG-PCL with block lengths of PEG-PCL—5,000:5,000 (CL5) and 5,000:15,000 (CL15) were prepared as described above in detail for the PEG–PLA di-block.

*Characterization of the PEG-PCL and PEG-PLA di-block*

The ^1^H NMR (Bruker Avance 400 MHz NMR spectrometer (Germany)) spectra of the PEG-PCL and PEG-PLA di-block were measured in CDCl3 at room temperature. Agilent GPC-Addon apparatus GPC-Addon apparatus with Plgel® columns was used to determine the molecular weights and polydispersity of prepared di-block. Tetrahydrofuran as an eluent and polystyrene standards as a calibration were used.

**Table 1:** **Copolymer of PEG-PLA characteristics determined by** **1** **HNMR and GPC**

**1** **H-NMR**			**GPC**		
**copolymer**	**Mn** **a**	**PLA/PEG** **b**	**Mn** **c**	**Mw** **d**	**Mw/Mn** **e**
		**Real Theory**			
**LA5**	**8589.7**	**0.72 1**	**7872**	**9027**	**1.15**
**LA15**	**16383**	**2.28 3**	**7403**	**11999**	**1.62**

**a) Average Mn determined by 1H-NMR**

**b) PLA/PEG determined by 1H-NMR**

**c) Average Mn determined by GPC**

**d) Average Mw determined by GPC**

**e) Polydispersity identified using GPC**

**Table 2:** **Copolymer of PEG-PCL characteristics determined by** **1** **HNMR and GPC**

**1** **H-NMR**			**GPC**		
**copolymer**	**Mn** **a**	**PLA/PEG** **b**	**Mn** **c**	**Mw** **d**	**Mw/Mn** **e**
		**Real Theory**			
**CL5**	**11944**	**0.46 1**	**6926**	**7915**	**1.14**
**CL15**	**19492**	**2.9 3**	**9381**	**16228**	**1.73**

**a) Average Mn determined by 1H-NMR.**

**b) PCL/PEG determined by 1H-NMR.**

**c) Average Mn determined by GPC.**

**d) Average Mw determined by GPC.**

**E) Polydispersity identified using GPC** **.**

**Table 3:** **Micelles formulation contains DTX with different proportions of CL5 and LA5**

**No.**	**CL5%**	**LA5%**	**Z-Average (nm)**	**PdI**	**Zeta potential (mV)**	**DTX (µg/mL)**	**EE%**	**LC%**
**M1** **a**	**100**	**0**	**48.69 ± 4.2**	**0.445 ± 0.1**	**-6.14**	**114.7 ± 0.1**	**57.35 ± 0.25**	**0.57 ± 0.04**
**M2** **b**	**0**	**100**	**36.19 ± 7.8**	**0.249 ± 0.04**	**-7.62**	**92 ± 0.02**	**46 ± 0.03**	**0.46 ± 0.02**
**M3** **c**	**50**	**50**	**31.36 ± 5.6**	**0.233 ± 0.09**	**-18.9**	**113 ± 0.35**	**56.5 ± 0.07**	**0.56 ± 0.07**
**M4** **d**	**25**	**75**	**36.4 ± 6.8**	**0.344 ± 0.05**	**-16.6**	**100 ± 0.02**	**50 ± 0.62**	**0.50 ± 0.01**
**M5** **e**	**75**	**25**	**32.65 ± 8.3**	**0.262 ± 0.02**	**-12.7**	**174 ± 0.03**	**87 ± 0.09**	**0.87 ± 0.09**

**a) M1: Micelle contains 100 % CL5 and 0 % LA5.**

**b) M2: Micelle contains 0 % CL5and 100 % LA5.**

**c) M3: Micelle contains 50 % CL5 and 50 % LA5.**

**d) M4: Micelle contains 25 % CL5 and 75 % LA5.**

**e) M5: Micelle contains 75 % CL5 and 25 % LA5.**

**Table 4:** **Polymerosomes formulation contains DTX with different proportions of CL15 and LA15**

**No.**	**CL5%**	**LA5%**	**Z-Average (nm)**	**PdI**	**Zeta potential (mV)**	**DTX (µg/mL)**	**EE%**	**LC%**
**M1** **a**	**100**	**0**	**48.69 ± 4.2**	**0.445 ± 0.1**	**-6.14**	**114.7 ± 0.1**	**57.35 ± 0.25**	**0.57 ± 0.04**
**M2** **b**	**0**	**100**	**36.19 ± 7.8**	**0.249 ± 0.04**	**-7.62**	**92 ± 0.02**	**46 ± 0.03**	**0.46 ± 0.02**
**M3** **c**	**50**	**50**	**31.36 ± 5.6**	**0.233 ± 0.09**	**-18.9**	**113 ± 0.35**	**56.5 ± 0.07**	**0.56 ± 0.07**
**M4** **d**	**25**	**75**	**36.4 ± 6.8**	**0.344 ± 0.05**	**-16.6**	**100 ± 0.02**	**50 ± 0.62**	**0.50 ± 0.01**
**M5** **e**	**75**	**25**	**32.65 ± 8.3**	**0.262 ± 0.02**	**-12.7**	**174 ± 0.03**	**87 ± 0.09**	**0.87 ± 0.09**

**a) P1: Polymersome contains 100 % CL15 and 0 % LA15.**

**b) P2: Polymersome contains 0 % CL15 and 100 % LA15.**

**c) P3: Polymersome contains 50 % CL15 and 50 % LA15.**

**d) P4: Polymersome contains 25 % CL15 and 75 % LA15.**

**e) P5: Polymersome contains 75 % CL15and 25 % LA15.**

**Table 5:** **The kinetic profiles of drug release from the micelles**

			** Release model**			
			**Higuchi**		**Zero-Order**	
**No.**	**CL5%**	**LA5%**	**Slope**	**R** **2**	**Slope**	**R** **2**
**M1**	**100**	**0**	**0.4982**	**0.9879**	**0.0456**	**0.9663**
**M2**	**0**	**100**	**0.9125**	**0.989**	**0.0834**	**0.9659**
**M3**	**50**	**50**	**0.4846**	**0.9882**	**0.0442**	**0.9608**
**M4**	**25**	**75**	**0.537**	**0.9887**	**0.049**	**0.9639**
**M5**	**75**	**25**	**0.3906**	**0.9887**	**0.0356**	**0.9616**

**Table 6:** **The kinetic profiles of drug release from the polymersomes**

			**Release model**			
			**Higuchi**		**Zero-Order**	
**No.**	**CL15%**	**LA15%**	**Slope**	**R** **2**	**Slope**	**R** **2**
**P1**	**100**	**0**	**0.5055**	**0.9936**	**0.0462**	**0.9403**
**P2**	**0**	**100**	**0.7876**	**0.99**	**0.0714**	**0.9243**
**P3**	**50**	**50**	**0.6015**	**0.9931**	**0.0548**	**0.9358**
**P4**	**25**	**75**	**0.6957**	**0.9935**	**0.0635**	**0.939**
**P5**	**75**	**25**	**0.3605**	**0.9924**	**0.0328**	**0.9334**

**Table 7:** **IC** **50** **values (µg/mL) for commercial DTX formulation (Taxotere®), DTX-loaded micelles (M5) and polymersomes (P5) on AGS, B16F10, MCF-7 and PC3 cells**

**Cell-Line**								
	**AGS**		**B16F10**		**MCF7**		**PC3**	
**IC** **50 ** **(µg)**	**24 h**	**48 h**	**24 h**	**48 h**	**24 h**	**48 h**	**24 h**	**48h**
**P5-DTX**	**11.79**	**5.494**	**9.47**	**8.75**	**3.466**	**3.314**	**1.486**	**3.083**
**M5-DTX**	**7.239**	**7.085**	**21.67**	**12.319**	**11.72**	**10.499**	**7.778**	**5.920**
**DTX**	**13.06**	**12.70**	**21.64**	**1.022**	**1.375**	**1.08**	**11.938**	**11.092**

**Figure 1:**
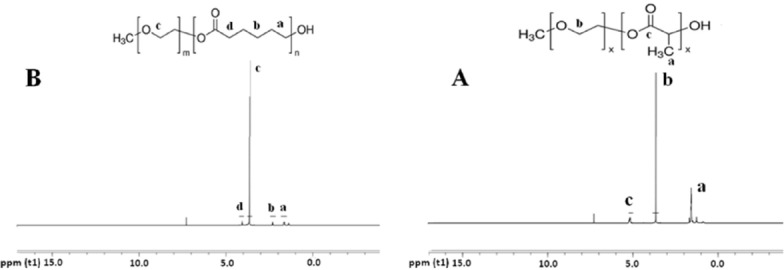
**The** **1** **HNMR spectra of the PEG-PLA (A) and PEG-PCL (B).**

**Figure 2:**
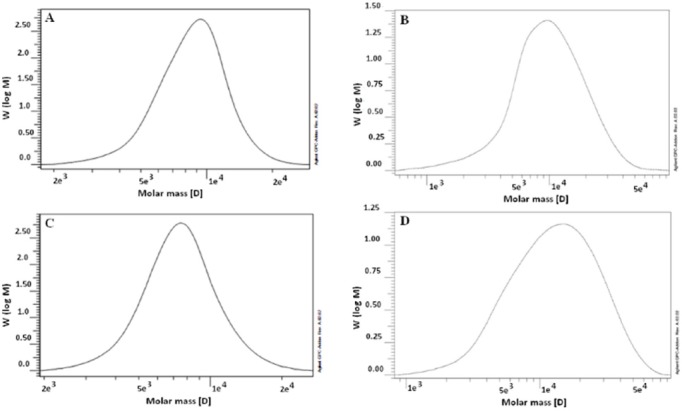
**GPC chromatogram of PEG-PLA 5000-5000 (A), PEG-PLA 5000-15000 (B) PEG-PCL 5000-5000 (C), and PEG-PCL 5000- 15000 (D)**

**Figure 3:**
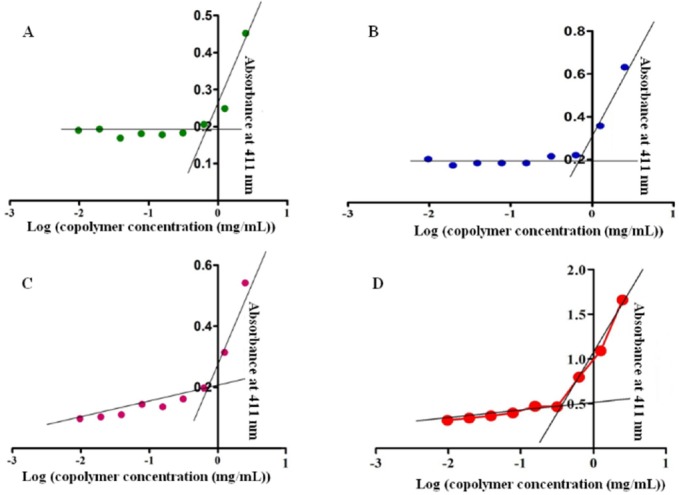
**Critical micelle concentration (CMC) of LA5 (A), LA15 (B), LA5 (C) and CL15 (D)**

**Figure 4:**
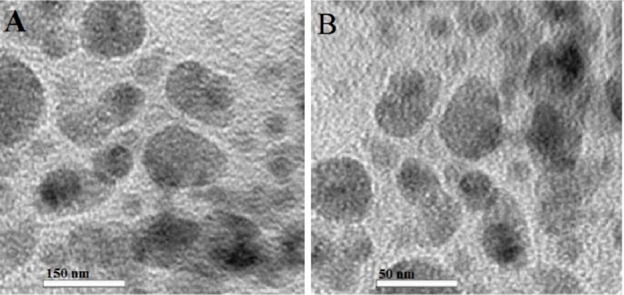
**TEM images of DTX-loaded micelles of M5 (A) and DTX-loaded polymersomes of P5 (B)**

**Figure 5:**
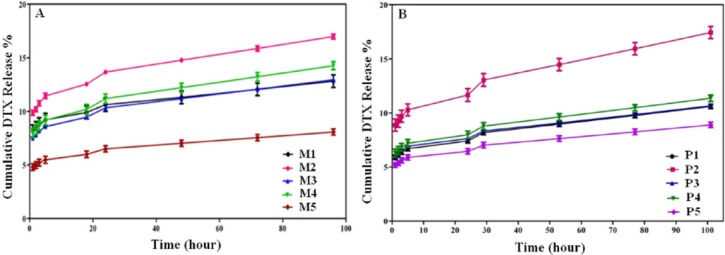
**Release curve of DTX from formulations in PBS pH 7.4 of mixed micelles (A) and polymersomes (B)**

**Figure 6.**
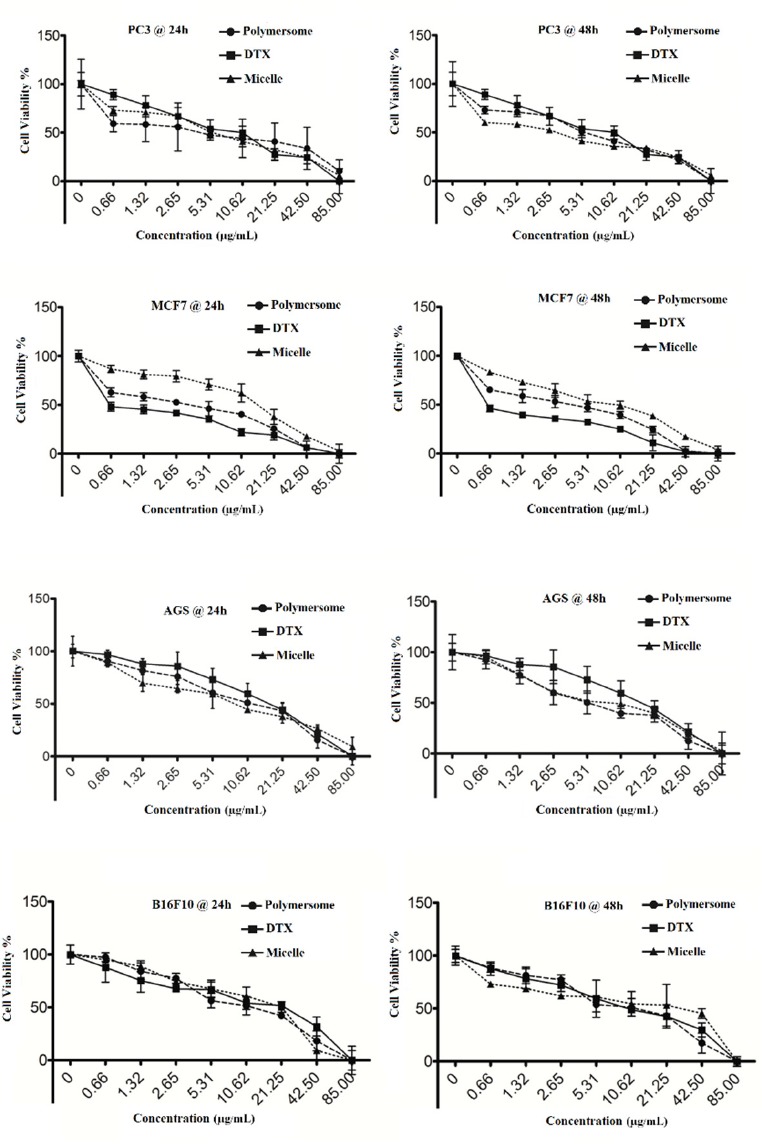
**Cytotoxic effect of polymersomes, DTX and mixed micelles against B16F10, PC-3, AGS and MCF-7 cells**

**Figure 7:**
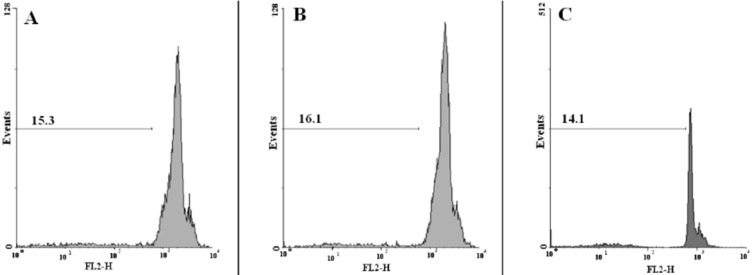
**Flow cytometry histograms of prostate cancer cells treated with polymersome (without DTX) (A), micelle (without DTX) (B),and control (C)**

**Figure 8:**
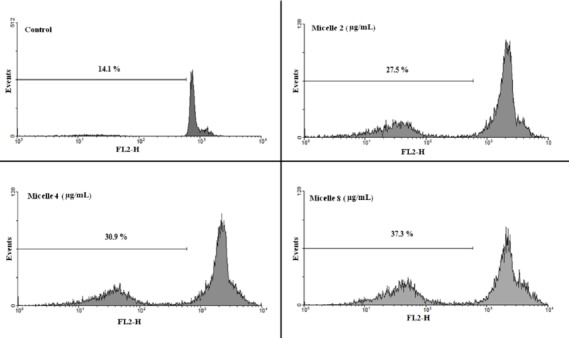
**Flow cytometry histograms of prostate cancer cells treated with different concentration of micelles**

**Figure 9:**
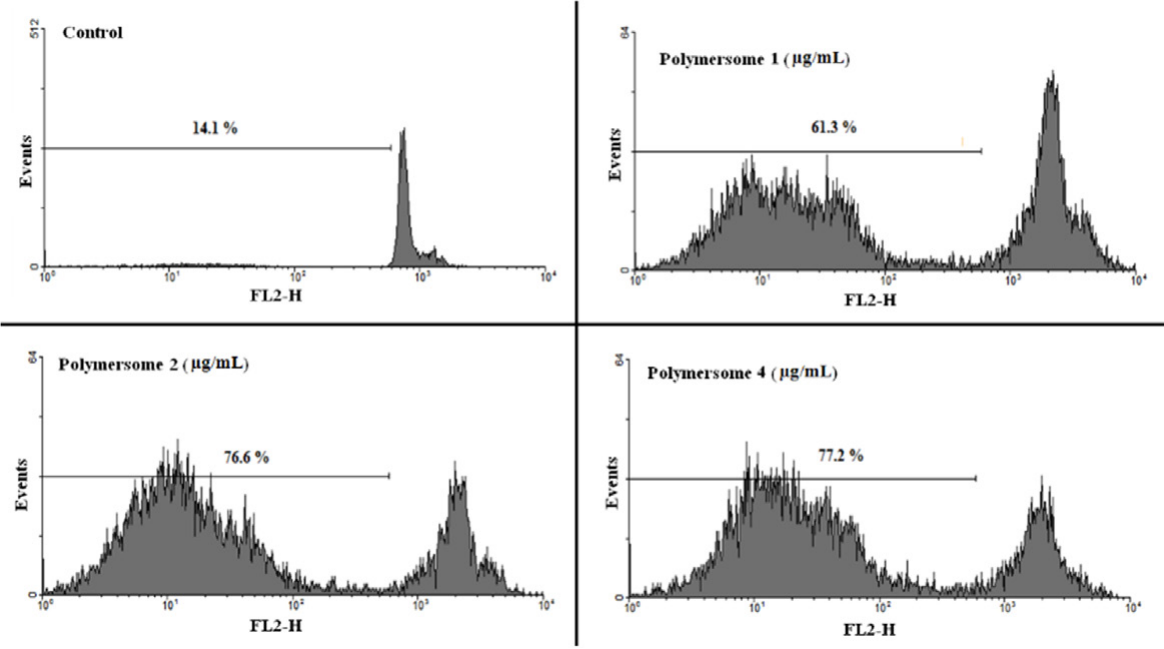
**Flow cytometry histograms of prostate cancer cells treated with different concentration of polymersomes**

**Figure 10:**
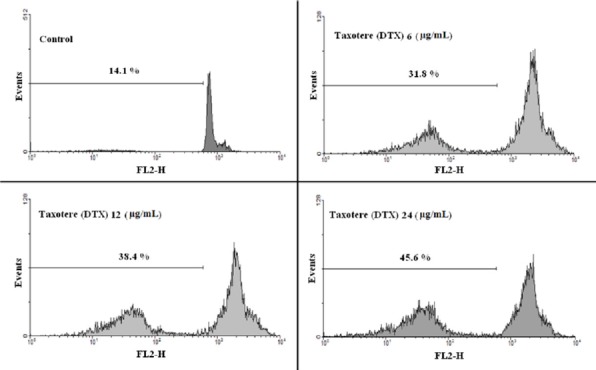
**Flow cytometry histograms of prostate cancer cells treated with different concentrations of Taxotere (DTX)**

*Preparation of DTX-Loaded di-block*

Single emulsion-solvent evaporation technique was used to prepare the PEG-PLA and PEG-PCL mixed nanoparticles (micelles and polymersomes) with different proportions, including 0:1, 3:1, 1:3, and 1:0. First, 5 mg of docetaxel powder was added in 5 mL of tetrahydrofuran. Then, 20 mg of di-block was dissolved in 2 mL of tetrahydrofuran using sonicator. Next, 200 µL of the DTX solution was loaded to the di-block solution with stirring. The emulsion was prepared by adding the dropwise of mixture to 6 mL of deionized water in a baker with stirring for 4 h. Rotary evaporator was used to eliminate the organic solvent under reduced pressure to form the nanoparticles. Finally, the free DTX was separated from NPs by ultrafiltration in Amicon centrifugation filters (15 min, 4500 rpm, cut off: 3500 Da).

*Physiochemical Properties of self-assembled structure*

Dynamic light scattering (DLS) (Malvern Zetasizer ZS; Malvern, UK) was used to detect the particle sizes and size distributions of self-assembled in distilled water (25 °C) at scattering angle of 90° using He-Ne laser at 633 nm incident beam. Furthermore, Transmission electron microscopy (TEM) (Hitachi H-7000, Nissei Sangyo, USA) confirmed the size and homogeneity of the micelles and polymersome via DLS. In practice, a dilute suspension of copolymers in distilled water (0.5 mg/mL) had filtered through a 450 nm filter on copper grids covered with a carbon film and observed at 80 kV.

*Critical Micelle Concentration Measurement*

Modified dye solubilization method was assessed the critical micelle concentration (CMC) of the polymersomes and micelles in distilled water. In this method, the aliquots of iodine solution in acetone (500 µL from 11.25 mg/mL) instead of pyrene (15, 16) were added to different concentration of copolymer suspension (0.001-2.5 mg) in distilled water. The CMC can be verified by an abrupt increase in the absorbance measurement using UV–vis spectra (UV-160A Shimadzu) at λ_max_=411 nm. 

*Drug loading content and encapsulation efficiency*

The DTX concentration in the micelles and polymersomes was analyzed via HPLC (Acme 9000 Young Lin, South Korea) through C-18 ODS columns (4.6 × 250 mm2) using UV-detector at

λ = 230 with mobile phase containing acetonitrile/water (55: 45, v/v), flow rate 1.0 mL/min, an injection volume of 20 µL at room temperature. The DTX-loaded NPs (100 µg) were dissolved in 900 µL of acetonitrile. The amount of DTX encapsulated in the self-assembled structure was measured using standard calibration curve. The drug loading content (LC) and encapsulation efficiency (EE) of the micelles and polymersomes were measured by using the formulas (1-2): 

 Equ 1 $$\mathrm{LC\%}=\frac{\mathrm{Mass of DTX in the formulation}}{\mathrm{Mass of copolymer in the formulation}}$$

Equ 2 $$\mathrm{EE\%}=\frac{\mathrm{Mass of DTX loaded in the sample}}{\mathrm{Mass of DTX initially used}}$$

*In-vitro Drug Release Studies*

Dialysis method under sink conditions in phosphate buffer saline (pH 7.4) composed of 0.1% Tween 80 was used for the *in-vitro* drug release studies of formulations. The DTX-loaded formulations (1 mL) were added to a dialysis bag (MWCO 1.2 kDa) and then were incubated at 90 rpm and 37 °C. At definite time intervals, 1mL of the release medium was withdrawn to evaluate DTX concentration using HPLC, while 1 mL of fresh release medium replaced to maintain sink conditions. 

*Cell Culture*

The AGS (human Caucasian gastric adenocarcinoma cell line), MCF-7 (human breast carcinoma cell line), B16F10 (mouse melanoma cell line), and PC-3 (human prostate carcinoma cell line) cells were purchased from the Pasteur Institute of Iran. The growth condition of the cells were in RPMI 1640 medium (Gibco BRL, USA) containing 10% (v/v) heat-inactivated fetal bovine serum (FBS) (Gibco BRL, USA), 100 U/mL of penicillin, 100 units/mL in an atmosphere containing 5% CO_2_ at 37 °C. 

*Cytotoxicity Studies*

The AlamarBlue^®^ assay was applied to study the cytotoxicity of the commercial DTX formulation (Taxotere^®^), blank or DTX-loaded micelles (PEG-PCL/PEG-PLA 3:1) and polymersomes (PEG-PCL/PEG-PLA 3:1) against different cell lines at eight different concentrations. 

The cells were seeded at 10000 cells per well into 96-well plates. Then, the cells were incubated with 100 µl of formulations so that the final volume in each well was 200 µL and the final concentrations were 0.8, 1.25, 2.5, 5.5, 10.0, 21.0, 42.0, and 84.0 µg/mL for 24 h and 48 h. After that, 20 µL of AlamarBlue^®^ incubated in each well for 4 h and the absorbance at wavelengths of 570 and 600 nm was recorded using microplate reader. The percent of viability was calculated for each well.

*Apoptosis studies*

Propidium iodide (PI) staining and flow cytometry of the treated cells were used to determine the apoptotic cells and detect the sub-G1 peak, respectively (15, 16).

DNA fragmentation produces small fragments of DNA that can be eluted following incubation in a hypotonic phosphate–citrate buffer. When marked with a quantitative DNA-binding color such as PI, the cells that have missing DNA will take up less color and will appear to the left of the G1 peak. Summarily, the cells were seeded at 10^3^ cells per well into 24-well plates and incubated for 48 h with different concentrations of the commercial DTX formulation (Taxotere^®^), blank (NPs without DTX) or DTX-loaded micelles (PEG-PCL/PEG-PLA 3:1), and polymersomes (PEG-PCL/PEG-PLA 3:1). Floating and sticking cells were then collected and incubated at 4 °C overnight in the dark with 750 µL of a hypotonic buffer (50 µg/mL PI in 0.1% sodium citrate plus 0.1% Triton X-100) before flow cytometric analysis using a BD flow cytometer (Becton Dickinson, NJ, USA); the population of cells was calculated using the WinMDI software.

*Statistical Analysis*

All results shown represent means ± SEM from triplicate experiments performed in a parallel manner, unless otherwise indicated. Significant differences between groups were analyzed by one-way ANOVA. All comparisons were made relative to untreated controls, and the significance of difference is indicated as *P < 0.05, **P < 0.01, and ***P < 0.001.

## Results and Discussion

*Synthesis and Characterization of PEG-PLA and PEG-PCL Copolymers*

For all copolymers, the synthesis yield value was above 90%, which shows that synthesizing these copolymers by microwaves irradiation is simple, fast, highly efficient, limited production of by-products, and easier scale up that was confirmed by results of ^1^HNMR and GPC analysis (17-19). Figure 1 shows the ^1^HNMR spectrum of the PEG-PLA and PEG-PCL di-block. Methylene group of the PEG segment in the PEG-PLA was detected in the signal of 3.6 ppm. The signals of CH and CH_3_ of the lactide at the PLA segment are at 5.1 and 1.6 ppm, respectively as tabulated in Table 1. The average chain length of PLA and PEG can be estimate using their integral.

CH_2_ group of PEG segment in the PEG-PCL detected in the signal of 3.6 ppm. The signal of CH_2_ of the caprolactone next to the C = O at the PCL block was detected in 4.1 ppm. Other CH_2_ residues of the PCL block appeared at 2.3 and 1.6 ppm as multiples are tabulated in Table 2. The average chain length of PCL and PEG can be estimated using their integral. Molecular weights and polydispersity of di-block via GPC analysis also presented in Tables 1 and 2 that show a narrow polydispersity (< 2) for all of the di blocks. Figure 2 shows GPC chromatograms of the di-blocks, which are nearly a symmetric peak. The unimodal GPC curve with the low polydispersity of the di-block copolymers was verified.

*Preparation and Characterization of NPs*

Tables 3 and 4 present the results of dynamic light scattering of the PEG-PLA and PEG-PCL mixed nanoparticles, polymersomes (P1-P5), and micelles (M1-M5) loaded DTX with different proportions of CL5:LA5, including 1:0, 0:1, 3:1, and 1:3. Micelles are tiny droplets that are formed in these copolymer mixtures when the PEG segment of the copolymer is in contact with the water and the PLA and PCL repels the water. However, in CL15, LA15, and different proportions of these copolymers described above, the hydrophobic, long, and bulky segment of PLA and PCL tends to form a bilayer surface of polymersomes.

Therefore, the PLA and PCL segments are towards the inside of the bilayer and the hydrophilic PEG segments face the water molecules. Thus, these di-block copolymers form a vesicle structure (20).

Tables 3 and 4 show that the particle sizes of the micelles and the polymersomes were 30–50 nm and 140–180 nm, respectively. The particle dispersion indexes (PdI, 0.15–0.4) also showed that the nanoparticles had an acceptable size distribution and negative zeta potential that may stabilize micelles and polymersomes.

The CMC values of LA5, LA15, CL5, and CL15 were 0.625, 0.625, 0.625, and 0.312µg/mL, respectively (Figure 3). Thermodynamic stability caused by low CMC of the nanoparticles was prepared from these copolymers and could maintain micelle and polymersomes forms upon dilution.

The size of the micelle and polymersome containing DTX measured via TEM (Figure 4) was approximately 50 and 150 nm respectively, confirmed by dynamic light scattering (DLS) results (Tables 3-4). These results indicate the shape of formulations were uniform.

*In-vitro Drug Release Studies*

The core of micelles or the shells of the polymersomes could encapsulate the part of hydrophobic of DTX. The loading content and encapsulation efficiency for different micelle (M1-M5) and polymersome (P1-P5) formulations are presented in Tables 3 and 4.

The larger sizes of the polymersomes (~150 nm) showed higher loading content in comparison to micelles (~50 nm). Tables 3 and 4 show that the micelles and polymersomes of 75% of PEG-PCL (M5 and P5) have maximum DTX-loaded levels of 0.87% and 0.93%, respectively, and the micelles and polymersomes of 100% PEG-PLA (M2 and P2) have minimum DTX-loaded levels of 0.46% and 0.66%, respectively.

The CL segment of the copolymer PEG-PCL is more hydrophobic that of PEG-PLA, and so the hydrophobic drug DTX tends to accumulate more in the PEG-PCL copolymer. As a result, by increasing the ratio of PEG-PLA to PEG-PCL in the copolymer mixture, the loaded DTX was reduced so that a 100% PEG-PLA formulation (M2 and P2) showed the least loading amount of DTX (0.46 for M2 and 0.66 for P2) (21).

In contrast, we expected to have the highest LC for the 100% PEG-PCL formulation (M1 and P1). However, it was observed that the loaded DTX is enhanced by increasing the ratio of PEG-PCL up to 75% (0.87 for M5 and 0.93 in P5), and in 100% of PEG-PCL, the loaded DTX was decreased (0.57 for M1 and 0.71 for P1). Perhaps the more polar PLA segment is capable of forming hydrogen bonds with the polar hydroxyl and ketone groups in the DTX molecule. We conclude that there should be an optimum balance between hydrophobicity and hydrophobicity.

The *in-vitro* drug release curves of formulations were compared in PBS (pH 7.4) containing 0.1% Tween 80. The results are presented in Figure 5. The amounts of drug released in the 96-hour duration from micelles and polymersomes were 8–18% and 7–17%, respectively, which showed a sustained release pattern of both the micelles and polymersomes, indicating that our formulation method is suitable for a nano structure sustained drug delivery system. Figure 5 show that the micelles and polymersomes of 75% of PEG-PCL (M5 and P5) have the least amount of release, and the micelles and polymersomes of 100% PEG-PLA (M2 and P2) have maximum amount of DTX-release in the 96-hour period compared to the other copolymers.

The initial burst release of DTX from the micelles and polymersomes was greater than 5 to 10% within the 24 h due to the DTX adsorbed on the surface or near the surface of NPs (22). After that, a profile of DTX release reached a plateau trend in which approximately 8 to 18% of the loaded drug within 4 days, which shows the sustained release of DTX, is related to the DTX diffusion or di-block copolymers erosion mechanisms (23).

Micelles and polymersomes of 75% PEG-PCL and 25% PEG-PLA (M5 and P5 formulations) had the lowest burst release and the micelles and polymersomes of 100% PEG-PLA (M2 and P2) had the highest burst release in the first hour compared to other copolymers. Furthermore, as indicated, the M5 and P5 formulations have the most LC and EE. 

It seems the balance of 75% PEG-PCL to 25% PEG-PLA makes enough hydrophobicity for loading hydrophobic drugs such as DTX. Additionally, the 25% PEG-PLA enables the formulation to have the capability to build hydrogen bonds with the drug, improving drug encapsulation in this formulation. Therefore, drug release from this formulation may have the slowest pattern. 

The data of the kinetic profiles of drug release indicate that the micelles and polymersomes released DTX (Tables 5 and 6) according to the Higuchi model (Equ. 3) (18). The rate of DTX release in the first few hours was much higher, it can be concluded that the main mechanism of release was diffusion. After that time, the gradient concentration of DTX from all of the formulations became slow, and matrix erosion became the main release mechanism, according to the zero-order model (Equ. 4) (18):

$Q=2C\sqrt[{2}]{\mathrm{Dt}/}\pi$            $\mathrm{Q a}\sqrt[{2}]{t}$**                      Equ. 3 **

**Q=Kt                     Qαt                     Equ. 4**

Where t is time, D is the diffusion constant, C is the initial drug concentration, K is the constant, and Q is the mass flux.

*Cytotoxicity of Commercial DTX Formulation (Taxotere®), Blank (DTX-free formulation) or DTX-loaded Micelles (M5), and Polymersomes (P5) Against Different Cell Lines*

The data obtained from the AlamarBlue® assay indicated that the all of the formulations including M5 and P5 showed an increased anti-proliferative effect on B16F10, PC-3, AGS cells and MCF-7 cells as compared to Taxotere ®. The increased cytotoxicity in these cancer cells is considered a promising improvement in the therapeutic efficacy of DTX, when DTX is entrapped in the M5 and P5. Such a feature may be ascribed to a combination of higher DTX delivery to cancer cells and faster release of DTX from M5 and P5 within the cells. 

The DTX efflux from cancer cells, resulting in cancer resistance, could be evaded by nanoparticulation of DTX as they enter the cells via endocytotic pathway. Moreover, the metabolic activity of cancer cells which is usually higher than that of non-cancer cells may provide more acidic environment inside the cancerous cells resulting in M5 and P5 degradation, and consequently more DTX release and cytotoxicity. Accordingly, the increased DTX toxicity of N5 and P5 could be attributed to the higher uptake of DTX in the cancer cells. Furthermore, there is no significant difference between the release profiles of P5 and M5 formulations (Figure 5); therefore, the observed difference between cytotoxicity may be related to differences between cellular uptakes (24). In conclusion, the anti-proliferative effect of the formulations on cancer cells followed the order as P5~M5 > DTX. Additionally, the DTX-free formulation of both M5 and P5 manifested no cellular toxicity, confirming their non-toxic entity (Figure 6). Half maximal inhibitory concentration (IC_50_) values for various formulations against B16F10, PC-3, AGS, and MCF-7 cell lines are tabulated in Table 7.

## Conclusion

The combination of PEG-PLA and PEG-PCL 5000-5000 with different proportions, including 0:1, 1:1, 3:1; 1:3, and 1:0, formed micelles (M1-M5), and the combination of PEG-PLA and PEG-PCL 5000-15000 with the above ratios formed polymersomes (P1-P5). Application of these mixed micelles and polymersomes in encapsulating anticancer drug DTX was investigated to achieve an efficient formulation. Micelles and polymersomes of 75% PEG-PCL and 25% PEG-PLA (M5, P5) have the lowest burst release (5%) compared to the other copolymers (M1-M4, P1-P4). The polymersome P5 has the greatest cytotoxic effects among other formulation against B16F10, PC-3, and AGS cells, while MCF-7 cells were more sensitive to the cytotoxic activity of commercial DTX formulations compared to the other formulations. Treatment of PC-3 cells with formulations (P5, M5) significantly increased the sub-G1 peak, which is indicative of DNA fragmentation.
